# Mining Porcine Blood Whole-DNA Sequencing Datasets to Uncover Pig Viromes: An Exploratory Application to Identify Potential Infecting Agents of an Undefined Disease Outbreak

**DOI:** 10.3390/vetsci12060513

**Published:** 2025-05-24

**Authors:** Samuele Bovo, Anisa Ribani, Giuseppina Schiavo, Valeria Taurisano, Matteo Bolner, Francesca Bertolini, Luca Fontanesi

**Affiliations:** Animal and Food Genomics Group, Division of Animal Sciences, Department of Agricultural and Food Sciences, University of Bologna, 40127 Bologna, Italy; samuele.bovo@unibo.it (S.B.); anisa.ribani2@unibo.it (A.R.); giuseppina.schiavo2@unibo.it (G.S.); valeria.taurisano2@unibo.it (V.T.); matteo.bolner2@unibo.it (M.B.); francesca.bertolini3@unibo.it (F.B.)

**Keywords:** assay, genomics, next-generation sequencing, pathogen, post-weaning multisystemic wasting syndrome, *Sus scrofa*, virus

## Abstract

Pigs are affected by a variety of pathogenic agents that need to be identified correctly and diagnosed even when co-infections may complicate the application of specific and targeted assays. Next-generation sequencing can provide new perspective to monitor viruses infecting or co-infecting diseased pigs. In this study, we tested, for the first time for diagnostic purposes in a livestock species, a new method based on whole-genome sequencing of all the DNAs extracted from the blood of nine pigs sampled from a farm where there was a suspected outbreak of Post-weaning Multisystemic Wasting Syndrome. We then used unmapped reads on the porcine reference genome to mine for viral sequences using a specifically designed bioinformatic pipeline. Within this fraction of reads, viral sequences ranged from 0.002% to 4.4% of the total unmapped reads and were derived from twelve different viruses known to infect pigs, where three were herpesviruses, eight were parvoviruses, and one was a circovirus. All pig sequencing datasets were positive for one or more viruses, with various potential viral loads. Suid betaherpesvirus 2, also known as Porcine cytomegalovirus (PCMV), was the most frequently identified virus as five out of the nine pig sequencing datasets contained viral sequences from this virus. The results may suggest a heterogeneous viral profile of the diseased pigs that may be derived from potential secondary infections or co-infections. This pilot application demonstrated that a whole-genome sequencing approach can complement other routine diagnostic assays in veterinary virology. Other studies and improvements are needed to validate the results and apply this approach in routine monitoring applications.

## Simple Summary

Pigs can be infected or co-infected with numerous pathogenic and non-pathogenic viruses, making it challenging to interpret clinical symptoms. Diagnostic assays should provide unbiased results to help identify the infecting viruses. In this study, we tested a new and potentially unbiased method to identify viral sequences in porcine blood. The approach involves whole-genome sequencing of all extracted DNA from blood, isolating short DNA sequences not part of the pig genome (unmapped reads) and identifying viral sequences within this fraction. This method was used to investigate a suspected outbreak of Post-weaning Multisystemic Wasting Syndrome on a pig farm. By utilising advanced DNA sequencing technology and bioinformatic analysis of DNA sequencing data, we identified sequences from 12 different viruses within the sequencing data of nine diseased pigs from the farm. The results suggest a heterogeneous profile among the analysed pigs, potentially indicating potential secondary infections or co-infections. Considering the results of this pilot application, mining unmapped reads from whole-genome sequencing data can provide useful information to better understand complex viral disease states, which can help improve veterinary surveillance.

## 1. Introduction

Livestock species are affected by a variety of pathogenic agents that can cause similar symptoms. In certain environmental conditions or physiological states, many agents are difficult to detect, complicating their diagnosis or identification even when assays are available and correctly applied. Furthermore, assays for many pathogens are not commercially available. Additionally, newly emerging or re-emerging diseases are not yet included in any target assays or are caused by multiple agents, making it challenging to identify diseased animals and monitor their epidemiology and diffusion. All these conditions may create gaps in the ability to detect and identify animal diseases quickly and accurately. This is especially true for viruses that mutate rapidly and frequently cause complex syndromes derived from multiple co-infections, emphasising the need for suitable and comprehensive diagnostic methods [1,2]. It is also evident that viruses are major contributors to animal health emergencies, as demonstrated by recurrent outbreaks of viral diseases in pigs, such as foot and mouth disease, African swine fever, classical swine fever, porcine reproductive and respiratory syndrome (PRRS) and several emerging zoonotic diseases, among many others, causing concerns and large economic losses [3,4]. Therefore, monitoring viruses and being capable of correctly diagnosing viral diseases are essential for ensuring proper management actions to reduce their negative impacts at the pig farm level as well as within the pig production industry as a whole [4].

Pig viral diseases have traditionally been diagnosed using isolation systems; immunoassay-based methods, such as the enzyme-linked immunosorbent assay (ELISA); and targeted PCR assays or similar nucleic acid amplification-based methods [5]. Recently, high-throughput approaches have been proposed to investigate the pig virome by using next-generation sequencing for viral metagenomic analyses [6,7,8,9,10,11,12,13].

Viral metagenomics relies on specific nucleic acid extraction protocols from available specimens that are capable of enriching the isolated material of nucleic acids from viruses [14,15,16]. Some protocols include initial steps for the purification of viral particles, the removal of host nucleic acids and sequence-dependent or -independent amplification of viral nucleic acids (including the common sequence-independent single-primer amplification approach), followed by next-generation sequencing after library preparation and finally the identification of viral sequences by comparing obtained reads against annotated viral sequence databases, with several modifications and improvements in the flow of the various steps listed [17,18,19]. Despite viral metagenomics largely contributing to enhancing our understanding of the composition, diversity and pathogenic roles of animal viromes, the various ways in which different steps are conducted, starting from the viral extraction methodologies from different tissues to the amplification protocols, can produce biases in the obtained viral profiles [19,20,21,22,23,24].

Unbiased approaches not based on the enrichment of viral particles or amplification steps have been proposed to create catalogues of animal tissue-specific viromes. For example, the human blood DNA virome has been explored from whole-DNA sequencing (or whole-genome sequencing) data using sequence reads that did not map to the human reference genome [25,26,27]. A fraction of these unmapped reads was from viruses infecting the sampled individuals, demonstrating that mining whole-genome sequencing data obtained for other purposes (e.g., analysis of the host genome) can be useful for monitoring infections. We applied a similar approach by mining shotgun sequencing datasets obtained from blood DNA pools derived from 100 archived porcine samples to analyse the pig genome and, as a byproduct, identify several viruses of the Parvoviridae family [28]. Subsequently, we further expanded this approach by mining whole-genome sequencing datasets obtained to analyse the cattle, pig, chicken and rabbit genomes and identified a large number of viral sequences from the unmapped reads [29]. This demonstrates the utility of this approach in describing the animal DNA virome with potential diagnostic purposes, especially when the pathogenic condition is not clear and cannot be easily diagnosticated.

In this study, we tested this approach to search for a potential explanation for a disease outbreak that occurred on a pig farm. To our knowledge, this is the first practical pilot application of this approach to detect potential infecting agents in a livestock species. The animals investigated displayed symptoms that could be attributed to Post-weaning Multisystemic Wasting Syndrome (PMWS) or uncharacterised multi-virus infections. To evaluate the presence of undefined viruses, we used porcine blood as a source of DNA. The whole DNA was sequenced using a shotgun next-generation sequencing approach. We then used unmapped reads on the pig reference genome, which may contain sequence data not derived from the host genome, to identify virus sequences.

## 2. Materials and Methods

### 2.1. General Disease Description and Standard Laboratory Diagnostic Analyses

In May 2023, a disease outbreak occurred at a growth-to-finish pig farm (open-cycle) located in Northern Italy. The farm was closely monitored by veterinary personnel who provided clinical information. The affected animals, specifically seven-week-old pigs (about three weeks after weaning), displayed severe clinical signs, such as fever, dyspnea and coughing. The animals also experienced weight loss, gradually becoming emaciated, and unfortunately did not recover. Approximately 20–30% of the animals died one/two weeks after clinical signs appeared. Post-weaning Multisystemic Wasting Syndrome (PMWS) was suspected.

Before an experimental design for this study could be implemented, during the outbreak, veterinary inspectors collected lung specimens from four deceased pigs and sent samples to detect the presence of potential viral pathogens associated with respiratory and wasting syndromes in pigs. Diagnostic testing was conducted at Vaxxinova GmbH Diagnostic (Leipzig, Germany), where standard real-time PCR analyses were used to search for several viruses, including porcine circovirus 2 (PCV2), porcine circovirus 3 (PCV3), porcine reproductive and respiratory syndrome virus (PRRSV), and Swine influenza virus A (SIV). These analyses were conducted to monitor the presence of these viruses in the farm production sites.

Animals were not specifically bred, raised or treated for this study. The pig farm was closely inspected by authorised veterinary personnel as part of the routine husbandry and health management practices; therefore, no ethical concerns are applicable.

### 2.2. Sampled Pigs and Whole-Genome Sequencing

To further investigate the potential involvement of DNA viruses in the outbreak other than those tested directly, we applied a whole-DNA sequencing approach, expanding on the proof-of-concept demonstrated in our previous studies [28,29]. Whole-DNA sequencing analyses were applied to DNA samples obtained from nine pigs (specimens S1–S9) with the following characteristics: (i) the pigs were weaned at 28 days of age, (ii) vaccinated for PRRS at ~14 days, (iii) vaccinated for PCV at ~25 days and (iv) raised in three different sites (site #1, site #2 and site #3) of the farm (3 pigs/site) at ~50 days of age. Pigs were sampled for blood as part of the outbreak investigation by authorised veterinary personnel at 70–90, 50–80 and 95–105 days of age for site #1, site #2 and site #3, respectively. DNA was extracted using the PureLink™ Genomic DNA Mini Kit (Thermo Fisher Scientific, Waltham, MA, USA). A total of 4.0 μg of extracted DNA from each of the nine pig samples was used for library preparation. Nine genomic libraries of 300–400 bp in size were constructed and sequenced on a BGISeq500 machine (BGI, Shenzhen, China), following the provider’s protocol. Approximately 56 Gb of sequenced paired-end reads of 150 bp in length was obtained for each sampled pig.

### 2.3. Mining Whole-Genome Sequencing Datasets for Virus Sequences

The characterisation of the virome followed the bioinformatics pipeline previously outlined by Bovo et al. [29]. Briefly, the workflow consists of three main steps:

1. The development of a comprehensive virus database was based on the NCBI Virus resource (https://www.ncbi.nlm.nih.gov/genome/viruses; accessed on 5 February 2025), which contains DNA sequences of reference viral genomes and their strains. Processing and curating this resource are essential to create a reliable database.
2. Host-related reads were removed by aligning sequenced reads to the *Sus scrofa* genome, which was assembled using Sscrofa11.1. Unmapped reads, which represent non-host DNA, likely originating from microorganisms or viruses, were then extracted.
3. Screening for viral sequences involved aligning the extracted unmapped reads to the curated virus database to identify viral sequences. This process included a quick alignment using the BWA v.0.7.17 tool, followed by confirming alignments with a more sensitive approach, like the BLAST+ v.2.7.1 tool. Only viral genomes with a minimum of three aligned read pairs (six reads) were considered representative of the sample.

### 2.4. Statistical Analyses

Pearson’s correlation was utilised to compare the number of unmapped reads and the number of reads matching virus sequences across whole-genome sequencing datasets.

A positive match was defined as a dataset containing reads assigned to that specific virus. A dataset has the number of positive matches equal to the number of different viruses identified within its unmapped reads. Fisher’s exact tests were used to compare the number of positive matches across pigs from different production sites. This test aimed to assess potential differences in the number of distinct viruses infecting the three production sites.

Additionally, these tests were used to evaluate differences in virus infection loads between two pigs (from the same or different production sites) for the same virus. This was performed by comparing the corresponding pair of datasets and analysing the number of reads assigned to the identified virus in relation to the total number of unmapped reads. A significance threshold was set at *p* < 0.01 after Bonferroni correction for multiple testing.

A positive whole-genome sequencing dataset was defined as a dataset that contained reads mapping against at least one specific porcine virus. A positive dataset was also considered in relation to a specific virus. The number of positive datasets identified in this study was compared with the number of positive datasets identified by Bovo et al. [29] who mined a total of 464 porcine whole-genome sequencing datasets retrieved from the European Nucleotide Archive (ENA). These ENA datasets, derived from 52 pig populations from various countries, constitute random pig samples without any information on their health status. However, it may be assumed that they were from healthy populations based on the fact that these datasets were derived from animals that were declared to be healthy or for which the experimental analyses from which these datasets have been produced needed to be derived from healthy pigs [29]. For the comparison, Fisher’s exact tests were used. Also, for this group of tests, the significance threshold was set at *p* < 0.01 after Bonferroni correction for multiple testing.

## 3. Results

### 3.1. Description of the Disease Outbreak at the Farm Level and Standard Laboratory Analyses

As part of the preface of this study, we provide information on the routine veterinary practice that was initiated to evaluate the disease outbreak that occurred on the pig farm. Post-weaning Multisystemic Wasting Syndrome (PMWS) was initially suspected based on the clinical description of the affected piglets (which included fever, dyspnea, cough, weight loss and progressive emaciation), as documented by the veterinary personnel on the farm. Therefore, some specimens were collected by the veterinary personnel and submitted for routine investigation at a commercial veterinary laboratory. Given that PMWS may be primarily associated with PCV2 [30,31], specific diagnostic testing focused on detecting this virus and potential co-infecting agents [32,33,34,35], including PCV3, PRRSV and SIV. These tests were conducted on deceased animals. The four tested samples were negative for PCV2, suggesting that this virus may not be directly associated with the disease outbreak on this farm. Similarly, all four samples also tested negative for PCV3 and SIV, indicating that these viruses were not contributing to the observed clinical signs. Instead, PRRSV was detected in all samples, raising the possibility that this virus played a role in the outbreak. PRRSV is known to cause severe respiratory disease in post-weaning pigs, which may explain some of the symptoms observed in the affected animals.

### 3.2. Overview of Virus Sequences in Whole-DNA Sequencing Datasets

Since the preliminary viral profile obtained from deceased animals may not fully explain the observed clinical signs, we tested the possibility to obtain an unbiased screening of the blood DNA virome. This was performed by performing whole-DNA sequencing on the total DNA extracted from the blood of a select few affected pigs. These pigs were chosen randomly from those displaying the most severe clinical signs and were raised in three different production sites on the same farm (three pigs for each of the three sites). This approach targets DNA and cannot identify RNA viruses unless they utilise DNA intermediates during replication.

Whole-DNA sequencing was performed on each sample, generating approximately 400 million reads. On average, the *Sus scrofa* reference genome (host genome) had a coverage of 99%, with a sequencing depth of approximately 20×. Summary statistics of the unmapped reads are shown in Table 1. The average proportion of unmapped reads per sample was approximately 0.13% of all sequenced reads (Table 1). All the pigs studied had reads that matched some virus sequences. After further refinement of the alignments, the fraction of unmapped reads that truly mapped to viral genomes ranged from 0.002% to 4.40%. The correlation between the number of unmapped reads and the number of reads assigned to virus sequences was moderate (0.45). This suggests that there is a relationship between the content of DNA viruses and the total DNA not assigned to the pig genome, likely stemming from variations in virus infection loads among pigs.

### 3.3. Viral DNA Profiles in the Pigs Studied

A total of 25 matches with viral genomes, defined as the combination between pigs and the presence of reads from different viruses, were identified in the nine whole-DNA sequencing datasets (Table 2). Each match was confirmed by a minimum of three read pairs, with some viruses identified in multiple samples. Most of the pigs were positive for more than one virus, meaning that seven out of the nine obtained whole-DNA sequencing datasets contained reads matching two to six different viruses. Only two samples, one from site #1 (S2) and one from site #2 (S6), were positive for only one virus (the same in both samples: Suid betaherpesvirus 2).

**Figure 1:**
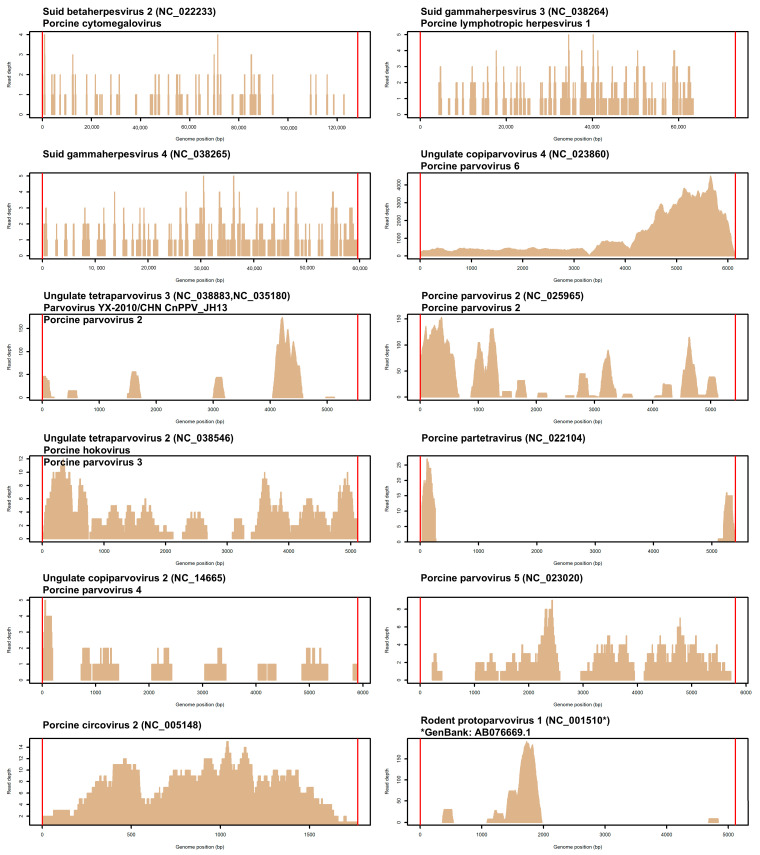
Graphical representation of the genome coverage of the 12 detected viruses. Vertical red lines denote the genomic boundaries, indicating the start and end of each genome.

These matches included a total of 12 different viruses (Table 2), all known to infect vertebrate species. The genome coverage for these viruses is shown in Figure 1. Among them, eleven viruses are clearly associated with pigs, where three are herpesviruses, seven are parvoviruses, and one is a circovirus. For two parvoviruses, reads consistently matched different Porcine parvovirus 2 (PPV2) reference genomes (Table 2), potentially identifying different strains. For this reason, we maintained separate information for each, counting them as separate viruses.

Among the identified herpesviruses, Suid betaherpesvirus 2 [SuBHV2; also known as Porcine cytomegalovirus (PCMV)] was the most frequently identified virus in terms of the presence of reads matching its genome among the sequenced reads of the investigated pigs. Five out of the nine pig samples from all production sites tested positive. Pig S2 had a significantly potential higher virus load compared to the other positive pigs (Table 2). This virus causes lifelong latent infections in pigs and can occasionally lead to rhinitis in piglets and reproductive disorders in pregnant sows [35,36]. Sequences from other herpesviruses were from Suid gammaherpesvirus 3 [SuGHV3; also known as Porcine lymphotropic herpesvirus 1 (PLVH1)] and Suid gammaherpesvirus 4 [SuGHV4; also known as Porcine lymphotropic herpesvirus 2 (PLHV2)]. These viruses are usually considered harmless or are associated with mild clinical signs [37,38]. Sequences matching Suid gammaherpesvirus 3 were identified in three pigs from two production sites (one from site #1 and two from site #3), with pig S9 from site #3 having a significantly higher load compared to the other two positive pigs. Sequences matching Suid gammaherpesvirus 4 were only found in one pig from site #2, with a relatively higher virus load compared to other herpesviruses in the other pigs (Table 2).

Among the identified parvoviruses, Ungulate copiparvovirus 4 [also known as Porcine parvovirus 6 (PPV6)] was the virus for which sequences were detected in three pigs, all from site #3. This virus has been associated with porcine reproductive failure in sows with mono-infections, although its pathogenic impact is still not completely clear [39]. Notably, the read counts supporting the detection of this virus were very high, reaching up to 4.4% of the unmapped read sets, with an average depth of over 1000 (Table 1 and Table 2). Pig S9 had a significantly lower virus load than the other two pigs.

Sequences matching four other parvoviruses were each identified in more than one pig: Ungulate tetraparvovirus 3, which had reads matching the reference sequence Parvovirus YX-2010/CHN CnPPV_JH13 and is considered a strain of Porcine parvovirus 2 (PPV2); Porcine parvovirus 2, with reads matching the Porcine parvovirus 2 strain BR/GO/ion_09_PPV-2/2011; Ungulate tetraparvovirus 2, also known as Porcine parvovirus 3 (PPV3) or Porcine hokovirus; and Porcine partetravirus. The two pigs (S1 and S4) that tested positive for both Ungulate tetraparvovirus 3 and Porcine parvovirus 2 had significantly different loads of viral reads. Since reads matching both viruses were identified in the same two pigs, it is possible that different strains of PPV2 may have infected the same animals, given the varying numbers of reads that were detected in the same animals for the two strains.

Reads from the other two parvoviruses, Ungulate copiparvovirus 2 [also known as Porcine parvovirus 4 (PPV4)] and Porcine parvovirus 5 (PPV5), were each identified in the sequencing dataset of only one pig.

Reads matching Porcine circovirus 2 (PCV2) (NCBI: NC_005148) were observed in one sample from site #1. PCV2 is the primary causative agent of porcine circovirus-associated disease (PCVAD) and the responsible primary agent of PMWS, including some enteric, respiratory and reproductive disorders [40].

Regarding the virus that was not directly associated with the pig, reads in two pigs (with significantly different viral loads) matched the version of the genome indicated to belong to Rodent protoparvovirus 1 (NCBI: NC_001510). However, reads better aligned with the GenBank sequence AB076669.1 rather than the indicated reference genome. This GenBank sequence has been included in an entry annotated as another parvovirus first identified in pigs from Myanmar [41]. Therefore, we can consider that all twelve different viruses may be regarded as prevalently associated with the pig, increasing the number of viruses assigned to the parvovirus group to eight, among those identified in the nine investigated pigs.

### 3.4. Putative Association of Virus Profiles with a Disease Condition

Based on the results regarding the prevalence of reads from different DNA viruses in the nine diseased pigs and the seemingly random distribution of these viruses, the results may suggest a heterogeneous profile that does not clearly explain the Post-weaning Multisystemic Wasting-like syndrome suggested by the clinical signs.

One out of the nine pig whole-DNA sequencing datasets contained reads from PCV2, which is usually associated with PMWS [30,31,32,33,34]. This virus was not detected in preliminary investigations that attempted to find explanations for the disease outbreak. The prevalence of the reads from this virus detected through whole-DNA sequencing (one out of the nine pigs) was lower than expected given the small number of pigs that was initially analysed as part of the preface of this study (none of the four pigs were positive for this virus).

We then evaluated whether there were some differences in the number of positive matches of viral sequences between the three production sites. Out of the 25 positive matches of viral sequences in the nine porcine whole-DNA sequencing datasets, 7 matches were found in pigs raised on site #1, while pigs raised in sites #2 and #3 had a total of 9 matches each (Table 2). There were no statistical differences in the number of positive matches between the three production sites.

In this study, we identified reads from seven viruses (Suid gammaherpesvirus 4, Suid gammaherpesvirus 3, Suid betaherpesvirus 2, Ungulate copiparvovirus 4, Ungulate tetraparvovirus 2, Porcine parvovirus 5 and Porcine partetravirus) that were also found in a previous study [29]. The previous study involved analysing 464 whole-DNA sequencing datasets from ENA derived from 52 different pig populations and many different studies. In this control dataset, Suid betaherpesvirus 2, which was the most common virus from which reads were identified in our current study (55.5%), had a frequency of 3.2%. The difference in positive datasets was statistically significant (Fisher’s exact test, *p* = 0.00009). Significant differences in prevalence between the current study and the reference datasets from ENA [29] were also observed for an additional five viruses: Suid gammaherpesvirus 3 (33.3% in this study and 13.1% in ENA; *p* = 0.0005), Ungulate copiparvovirus 4 (33.3% in this study and 1.3% in ENA; *p* = 0.0003), Ungulate tetraparvovirus 3 (22.2% in this study and 0.0% in ENA; *p* = 0.00032), Porcine parvovirus 2 (22.2% in this study and 0.0% in ENA; *p* = 0.00032) and Rodent Protoparvovirus 1 (22.2% in this study and 0.0% in ENA; *p* = 0.00032). Since all the pigs analysed in our study were diseased animals, the higher prevalence of reads from these viruses in diseased pigs may suggest a potential association with Post-weaning Multisystemic Wasting-like syndrome. Furthermore, a total of only 130 out of the 464 control datasets tested positive for at least one virus for which we detected reads in this study. The 100% detection rate of pigs in our group of diseased pigs was significantly different from the 28.0% observed in the ENA control datasets (*p* = 0.00001). This indicates that the presence of one or more of the detected viruses in pigs may be indicative of a potential association with a complex disease state.

## 4. Discussion

In this study, we tested the use of whole-DNA sequencing datasets obtained from diseased pigs as a potential source of viral sequence information for diagnostic purposes. This study can be considered a pilot application that can provide insights to evaluate the potential and limitations of this approach, with the final objective being to improve protocols and data analysis methodologies. Additionally, this exploratory approach may help clarify complex and undefined disease outbreaks caused by multiple pathogenic agents. Ultimately, this approach could complement other routine diagnostic assays in veterinary virology and traditional viral metagenomic analyses.

One advantage of the tested approach is that the production of whole-DNA sequencing datasets does not require any specific enrichment protocols of viral particles or amplification steps, eliminating the biases that may occur from these procedures. Standard DNA extractions are traditionally used to obtain DNA that is then sequenced. However, it is not yet clear whether different DNA extraction methods, protocols and kits may affect the possibility of capturing virus particles and producing viral sequences using this approach. A few studies have evaluated the potential impact of various DNA extraction kits and enrichment strategies on the outcomes of traditional viral metagenomic protocols, indicating that biassed results may be derived and could also be tissue-dependent [21,42,43]. However, these issues may be reduced in shotgun whole-DNA sequencing where all DNA is extracted and sequenced without any prefiltering or preference.

One of the main potential problems in viral metagenomics is the high frequency of contaminating virus sequences that are sometimes difficult to separate from the true viromes of the investigated specimens [16,44,45,46]. We did not identify any potential contaminating viral sequences that are commonly present in molecular-grade reagents or that are ubiquitous in some laboratories that routinely investigate viromes. This indicates that our laboratory procedures did not introduce any artefacts into the detected viral sequences.

Another advantage of shotgun whole-DNA sequencing is that novel DNA viruses are also sequenced, providing useful information to discover previously unknown viral diseases [25,26,27,28,29,47]. This should however be paired with specifically designed data mining bioinformatic pipelines that are capable of capturing novel viruses [47]. This may increase the computational effort in analysing the datasets. Computational efforts are already much higher in our approach than in traditional viral metagenomics because we first have to separate reads from those belonging to the host genome (the pig). We used only unmapped reads which are indirectly enriched with virus sequences. However, unmapped reads may also contain bacterial and fungal DNA derived not only from environmental contaminants but also from pathogenic agents, enlarging the possibility of using unmapped reads as a source of multi-kingdom pathogen information. We will further mine the datasets produced from the nine sampled pigs to obtain a more complete picture of the potential microbial agents contributing to the described disease state. Computational efforts may increase with the depth of sequencing. Increasing the depth of sequencing per sample (i.e., the number of reads generated) would also be useful to enhance the sensitivity of this approach in detecting viral sequences, as it is not entirely clear what the correlation may be between the viral load and the pathogenic conditions of the animals in most viruses. On the other hand, increasing the number of reads produced may also increase the cost of the assay. However, since the sequencing cost is continuously dropping and computing power is expected to increase, these conditions may make a routine approach based on whole-DNA sequencing at high depths feasible in the future. For comparative analyses across datasets or assays, it would be important to define some standards in terms of sequencing depth.

In this study, we analysed DNA sequencing datasets to identify DNA viruses. As a result, we were unable to detect any RNA viruses, which could be relevant in understanding the complex disease observed in the analysed pigs. PRRSV is an RNA virus that during the preface of this study has been shown to infect pigs of the farms from which we investigated the nine diseased pigs. To identify RNA viruses, one option is to sequence the entire transcriptome of a tissue/specimen and then analyse the obtained datasets to pinpoint porcine RNA viruses. Some studies have successfully utilised animal transcriptomes to identify RNA viruses using the same method we employed in our study by focusing on unmapped reads in the corresponding reference genome. This strategy has shown promise in identifying infections caused by RNA viruses [48,49]. However, this approach may increase costs not only for sequencing and data analysis but also for sampling to prevent RNA degradation. Another possibility is to simultaneously analyse DNA and RNA viruses by modifying nucleic acid extraction protocols and sequencing procedures, as suggested in viral metagenomics studies [50,51].

Another question arises from the tissue used for whole-DNA sequencing: blood. While blood may be the most convenient tissue to sample in live animals, it may not always be the most suitable target tissue for certain viruses. Some studies have evaluated this question applied to PMWS [52,53]. Sampling other tissues in deceased animals could provide a more accurate description of pathogenic infections. Conducting whole-DNA sequencing on multiple tissues could offer valuable comparative infection data, helping to identify the most appropriate tissue for specific diseases. However, analysing multiple tissues would increase the cost of the assay, making it unrealistic to routinely use more than one target tissue.

By mining whole-DNA sequencing datasets obtained from nine diseased pigs, we identified reads matching twelve viral genomes. There was no consistent viral profile among sites or pigs. In all diseased pigs, we found sequences for at least one pig-specific DNA virus. However, the number of positive matches for each pig varied from one to six different viruses, with a diversity among pigs suggesting that the identified DNA viruses could represent secondary infections or co-infections within another pathogenic condition primarily determined by PRRSV. Co-infection with multiple DNA viruses is common in PRRS, leading to synergistic pathogenicity and complex clinical signs [54,55,56]. A co-infection situation is also common in PMWS [6].

The number of reads identified for the same virus in different pigs may indicate varying viral loads. For some viruses, there were significant differences between pigs. For instance, reads matching Ungulate copiparvovirus 4 were found in all three pigs from site #3. In pig S9, the number of reads was approximately two orders of magnitude lower than that of the other two pigs. Significant variations in the number of virus reads between pigs were also observed for Suid betaherpesvirus 2, Suid gammaherpesvirus 3, Ungulate tetraparvovirus 3, Porcine parvovirus 2 and Rodent protoparvovirus 1. It will be interesting to validate these differences in viral loads with other analytical approaches, such as quantitative PCR.

To indirectly evaluate if the viral profile obtained for the nine investigated pigs could effectively be associated with their disease state, we used the results obtained in a previous study where we identified the presence of viral sequences in 464 porcine whole-DNA sequencing datasets retrieved from ENA as the reference [29]. These datasets have been produced from more than 50 pig populations that could indirectly be considered healthy. Seven out of twelve viruses (Suid betaherpesvirus 2, Suid gammaherpesvirus 3, Suid gammaherpesvirus 4, Ungulate copiparvovirus 4, Ungulate tetraparvovirus 2, Porcine parvovirus 5 and Porcine partetravirus) for which we detected reads in the nine diseased pigs were also present in some datasets derived from ENA. For three of these viruses (Suid betaherpesvirus 2, Suid gammaherpesvirus 3 and Ungulate copiparvovirus 4), there was a significantly lower prevalence in the ENA datasets than in the pigs analysed in this study. For the remaining four other viruses (Suid gammaherpesvirus 4, Ungulate tetraparvovirus 2, Porcine partetravirus and Porcine parvovirus 5), there were no statistically significant differences when considering their prevalence in the datasets of the nine investigated pigs and in the ENA dataset. Reads matching three other viruses (Ungulate tetraparvovirus 3, Porcine parvovirus 2 and Rodent protoparvovirus 1) were identified only in the nine pigs analysed in this study. Based on these comparative analyses, the presence of reads from five of these viruses (associated with the disease state, i.e., the three viruses with significantly higher prevalence in the diseased pigs of this study and the two viruses that were not present in the ENA dataset but were detected in the diseased pigs) may be indicative markers of a complex pathological condition in pigs similar to PMWS. However, this hypothesis should be considered with caution because the reference dataset was not specifically constructed for this purpose, and the precise health condition of the pigs in the 464 datasets is not completely known. A larger reference dataset with additional metadata per sample, sequenced in a standardised way, should be created to better facilitate comparative evaluations in defining potential diseased states in pigs for case and control studies, as we indirectly evaluated in this study.

## 5. Conclusions

To our knowledge, this exploratory study tested for the first time the possibility of using whole-DNA sequencing datasets as a source of viral information useful for detecting infection states in diseased pigs. For the routine application of this approach, some improvements are needed, specifically the ability to analyse both RNA and DNA viruses simultaneously. Additionally, comparing the viral profile in healthy pigs is necessary to interpret the viral profiles of diseased animals. Therefore, a larger dataset of whole-DNA sequencing data obtained in a standardised way would be necessary to avoid any biases in interpretation. Validation of the results derived from this approach with other viral metagenomic assays or specific target assays is needed to better evaluate the pros and cons of this approach. Looking ahead, mining unmapped reads from whole-DNA sequencing data can provide useful information to better understand disease states that are difficult to interpret with conventional assays and approaches.

## Figures and Tables

**Table 1:** Summary statistics on the whole-DNA sequencing datasets, including unmapped reads that matched virus sequences.

Pig	Site ^1^	Sequenced Reads	Unmapped Reads (%) ^2^	Reads of Viral Origin (%) ^3^
S1	#1	448,120,122	666,430 (0.15%)	934 (0.140%)
S2	#1	327,414,198	411,866 (0.13%)	44 (0.011%)
S3	#1	315,609,910	407,978 (0.13%)	10 (0.002%)
S4	#2	441,166,336	521,010 (0.12%)	378 (0.073%)
S5	#2	400,074,484	545,276 (0.14%)	84 (0.015%)
S6	#2	334,788,158	492,688 (0.15%)	22 (0.004%)
S7	#3	448,195,724	606,724 (0.14%)	26,720 (4.40%)
S8	#3	448,168,478	572,470 (0.13%)	14,618 (2.55%)
S9	#3	416,512,760	526,100 (0.13%)	572 (0.108%)

^1^ describes the production sites where animals were sampled. ^2^ represents the reads that did not align on the *Sus scrofa* reference genome (host genome). The percentage is given in relation to the sequenced reads. ^3^ represents the reads that aligned on the viral sequences in the virus database. The percentage is given in relation to the unmapped reads.

**Table 2:** The number of reads identified for the 12 viruses for the analysed pigs (S1–S9) raised in different production sites (#1–3) and the estimated depth of viral sequences on the corresponding reference viral genomes.

				Site #1 ^3^			Site #2 ^3^			Site #3 ^3^		
Virus Name	Alternative Name ^1^	NCBI ID	Depth ^2^	S1	S2	S3	S4	S5	S6	S7	S8	S9
Suid betaherpesvirus 2	Porcine cytomegalovirus (PCMV)	NC_022233	0.119	-	40 ^a^	-	22 ^b^	-	20 ^b^	12 ^b^	8 ^b^	-
Suid gammaherpesvirus 3	Porcine lymphotropic herpesvirus 1 (PLHV1)	NC_038264	0.172	-	-	6 ^a^	-	-	-	-	6 ^a^	72 ^b^
Suid gammaherpesvirus 4	Porcine lymphotropic herpesvirus 2 (PLHV2)	NC_038265	0.593	-	-	-	236	-	-	-	-	-
Ungulate copiparvovirus 4	Porcine parvovirus 6 (PPV6)	NC_023860	1027	-	-	-	-	-	-	26,616 ^a^	14,596 ^a^	496 ^b^
Ungulate tetraparvovirus 3	Parvovirus YX-2010/CHN CnPPV_JH13; Porcine parvovirus 2 (PPV2)	NC_038883; NC_035180	5.400	186 ^a^	-	-	10 ^b^	-	-	-	-	-
Porcine parvovirus 2	-	NC_025965	11.88	394 ^a^	-	-	36 ^b^	-	-	-	-	-
Ungulate tetraparvovirus 2	Porcine hokovirus (*p*-PARV4) PPV3	NC_038546	2.932	-	-	-	34	66	-	-	-	-
Porcine partetravirus	-	NC_022104	0.777	-	-	14	-	14	-	-	-	-
Ungulate copiparvovirus 2	Porcine parvovirus 4 (PPV4)	NC_014665	0.406	-	-	-	-	-	-	16	-	-
Porcine parvovirus 5	-	NC_023020	1.756	-	-	-	-	-	-	68	-	-
Porcine circovirus 2	-	NC_005148	6.441	76	-	-	-	-	-	-	-	-
Rodent protoparvovirus 1	Minute virus of mice	NC_001510	8.503	268 ^a^	-	-	24 ^b^	-	-	-	-	-

^1^ Alternative name or identified strain according to the information retrieved from the NCBI Viruses resource. ^2^ The estimated depth of viral sequences reported assumes an equal coverage of the reference genome. ^3^ Significantly different loads of viral reads for the same virus among different pigs are identified with different superscript letters (^a^, ^b^; *p* < 0.01 after Bonferroni correction for multiple testing).

## Data Availability

The sequencing datasets generated and analysed during the current study are available in the EMBL-EBI European Nucleotide Archive (ENA) repository (http://www.ebi.ac.uk/ena) under the study accession PRJEB87604.

## Abbreviations

The following abbreviations are used in this manuscript:

ELISA	Enzyme-linked immunosorbent assay
ENA	European Nucleotide Archive
Gb	Giga base pairs
PCV2	Porcine circovirus 2
PCV3	Porcine circovirus 3
PCVAD	Porcine circovirus-associated disease
PCVM	Porcine cytomegalovirus
PLHV1	Porcine lymphotropic herpesvirus 1
PLHV2	Porcine lymphotropic herpesvirus 2
PMWS	Post-weaning Multisystemic Wasting Syndrome
PPV2	Porcine parvovirus 2
PPV3	Porcine parvovirus 3
PPV4	Porcine parvovirus 4
PPV5	Porcine parvovirus 5
PPV6	Porcine parvovirus 6
PRRS	Porcine reproductive and respiratory syndrome
PRRSV	Porcine reproductive and respiratory syndrome virus
SIV	Swine influenza virus A
SuBHV2	Suid betaherpesvirus 2
SuGHV3	Suid gammaherpesvirus 3
SuGHV4	Suid gammaherpesvirus 4
